# 3D,2D-QSAR study and docking of novel quinazolines as potential target drugs for osteosarcoma

**DOI:** 10.3389/fphar.2023.1124895

**Published:** 2023-02-21

**Authors:** Zheng Lian, Chenglin Sang, Nianhu Li, Honglin Zhai, Wenzhe Bai

**Affiliations:** ^1^ School of Clinical Medicine, Weifang Medical University, Weifang, China; ^2^ Department of Orthopedics, The 960th Hospital of the Chinese People’s Liberation Army, Jinan, China; ^3^ The First Clinical Medical School, Shandong University of Traditional Chinese Medicine, Jinan, China

**Keywords:** quinazolines, structure-activity relationship, osteosarcoma, Targeted drugs, small molecule docking

## Abstract

**Background:** Quinazolines are an important class of benzopyrimidine heterocyclic compounds with a promising antitumor activity that can be used for the design and development of osteosarcoma target compounds.

**Objective:** To predict the compound activity of quinazoline compounds by constructing 2D- and 3D-QSAR models, and to design new compounds according to the main influencing factors of compound activity in the two models.

**Methods:** First, heuristic method and GEP (gene expression programming) algorithm were used to construct linear and non-linear 2D-QSAR models. Then a 3D-QSAR model was constructed using CoMSIA method in SYBYL software package. Finally, new compounds were designed according to molecular descriptors of 2D-QSAR model and contour maps of 3D-QSAR model. Several compounds with optimal activity were used for docking experiments with osteosarcoma related targets (FGFR4).

**Results:** The non-linear model constructed by GEP algorithm was more stable and predictive than the linear model constructed by heuristic method. A 3D-QSAR model with high Q^2^ (0.63) and *R* ^2^ (0.987) values and low error values (0.05) was obtained in this study. The success of the model fully passed the external validation formula, proving that the model is very stable and has strong predictive power. 200 quinazoline derivatives were designed according to molecular descriptors and contour maps, and docking experiments were carried out for the most active compounds. Compound 19g.10 has the best compound activity with good target binding capability.

**Conclusion:** To sum up, the two novel QSAR models constructed were very reliable. The combination of descriptors in 2D-QSAR with COMSIA contour maps provides new design ideas for future compound design in osteosarcoma.

## 1 Introduction

Osteosarcoma is a malignant tumor characterized by proliferating tumor cells directly producing bone or osteoid tissue, also known as osteosarcoma, derived from mesenchymal tissue (Ottaviani and Jaffe, 2009). Osteosarcoma is the most common primary malignant bone tumor with a high degree of malignancy, rapid growth and early metastasis. Early diagnosis of the disease is difficult and the prognosis is poor with an incidence of about 4–5/106 (Ottaviani and Jaffe, 2009; Moore and Luu, 2014). The average median age of diagnosis was 15 years, and the most common occurrence was between 15 and 20 years old, with 60% occurring below 25 years of age. Conventional osteosarcomas (classic) originate from the bone marrow and account for approximately 80% of all types of osteosarcomas, classified as osteogenic (50%), chondrogenic (25%), and fibrogenic (25%). Other rare osteosarcoma subtypes include capillary dilatation, small cell, parabone, periosteal, highly malignant surface osteosarcoma, low malignant central osteosarcoma, multi-center osteosarcoma and secondary osteosarcoma (Paget’s disease), etc. (Geller and Gorlick, 2010). Traditional osteosarcomas tend to occur in the long bones of the extremities, most commonly around the knee joint (distal femur, proximal tibia) (Sanerkin, 1980; Botter et al., 2014), with approximately 91% occurring in the metaphysis and 9% in the diaphysis. Atypical osteosarcoma can still invade non-long bones (skull, pelvis, mandible, vertebrae), and its incidence increases progressively with age. Common initial symptoms of osteosarcoma are pain and swelling, localized painful masses and inflammatory reactions, which may be followed by varying degrees of joint motion limitation and pathologic fractures.

At present, the treatment mode for osteosarcoma is preoperative neoadjuvant chemotherapy + surgical resection + postoperative adjuvant chemotherapy (Bishop et al., 2016). The main first-line chemotherapy compounds for osteosarcoma are methotrexate (MTX), doxorubicin (ADM), cisplatin (DDP), ifosfamide (IFO), vincritin (VCR), epirubicin (EPI), cyclo-phosphamide (CTX) and etoposide (VP-16), etc., in which the MTX, ADM, DDP and IFO are the most commonly used (Ta et al., 2009). However, in the current clinical work on osteosarcoma chemotherapy, these compounds were found to have several serious side effects, such as kidney damage caused by methotrexate and cisplatin, cardiac inhibition by doxorubicin, and resistance to chemotherapy compounds. Therefore, there is an urgent need to develop and design new and more effective compounds for the treatment of osteosarcoma.

Fibroblast growth factor receptor 4 (FGFR4) is a tyrosine kinase receptor that selectively binds to fibroblast growth factor 19 (FGF19). FGF19 binds to FGFR4 and its co-receptor b-Klotho, leading to dimerization and autophosphorylation of FGFR4. Activated FGFR4 interacts with fibroblast growth factor receptor substrate 2 (FRS2), recruits growth factor receptor binding protein (GRB2) and affects downstream proteins mediating osteosarcoma cell proliferation (Vainikka et al., 1994; Ho et al., 2009; Wu et al., 2011). Quinazoline derivatives exert an inhibitory effect on osteosarcoma growth by inhibiting the phosphorylation and signaling pathways of FGFR4 (Querolle et al., 2015; Nandi and Bagchi, 2016; Voskoboynik et al., 2016).

QSAR (Quantitative structure-activity relationship) is a compound research method that uses mathematical models to describe the relationship between the structure of a molecule and certain biological activities of the molecule. This method has been widely used for compound activity prediction and the development of new compounds (Dearden, 2017). Under the guidance of this method, people have successfully designed quinolones, such as norfloxacin. At present, the research methods of QSAR are mainly divided into 2D-QSAR and 3D-QSAR (Roy et al., 2015). Since 2D-QSAR research cannot accurately describe the relationship between molecular 3D structure and physiological activity, 3D-QSAR is more accepted by the scientific community in later compound design research. However, the effect of molecular descriptors in the 2D structure-activity relationships on the production of new compounds is often ignored when using a 3D method to design new compounds, which leads to unsatisfactory activity results of newly designed compounds. The main objective of this experiment was our desire to find a method that combines 2D-QSAR and 3D-QSAR to design more reliable compounds targeting osteosarcoma fibroblast growth factor receptor 4 (FGFR4).

## 2 Experiment

### 2.1 Data set

The data for this experiment included a total of 37 quinazoline derivatives, and all compounds were obtained from the references (Pan et al., 2021). The structures and activity values of all compounds are shown in Table 1.

**TABLE 1:** Structure and activity values of 37 compounds.

Structure	R_1_	R_2_	R_3_	IC_50_(nM)	Name
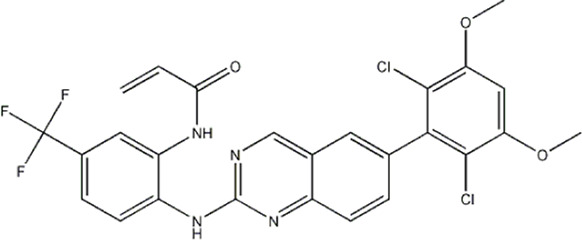	—	—	—	986	5a*
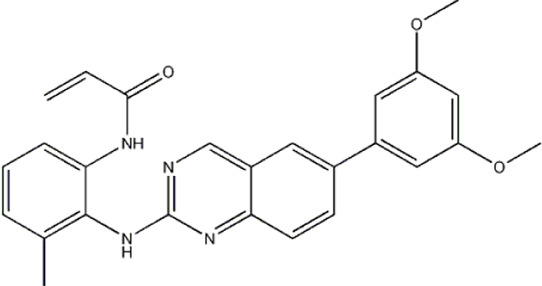	—	—	—	4011	5b*
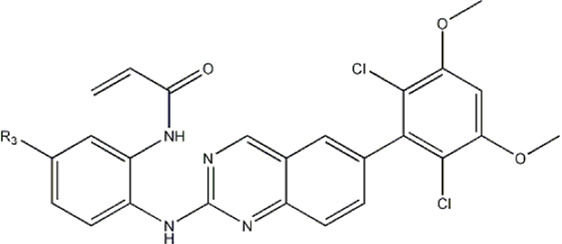	—	—	-Me	91	5c*
	—	—	-Et	268	5d
	—	—	-Pro	316	5e
	—	—	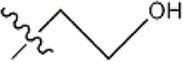	5.5	8a
	—	—	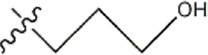	10	8b*
	—	—	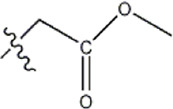	7.8	11a
	—	—	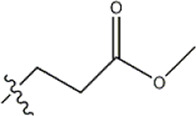	3.2	11b
	—	—	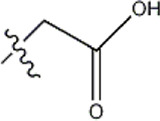	3.2	12a
	—	—	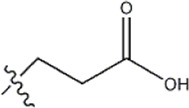	3.6	12b
	—	—	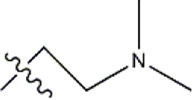	3.6	19a
	—	—	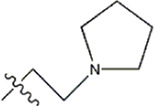	5.7	19c
	—	—	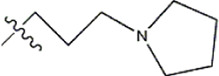	3.2	19d
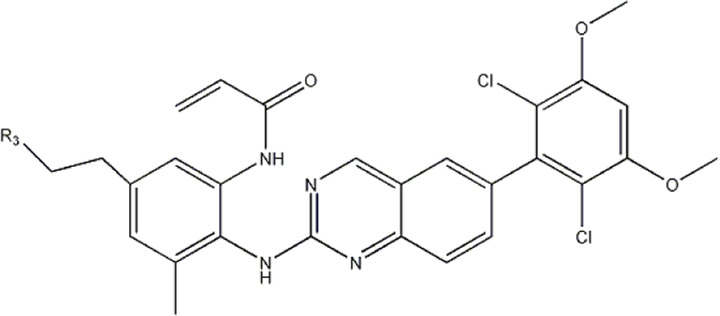	—	—	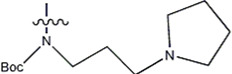	157	16a*
	—	—	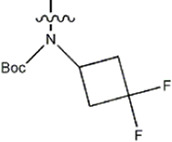	1.8	16b
	—	—	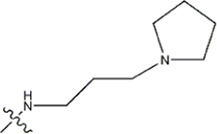	5.5	17a
	—	—	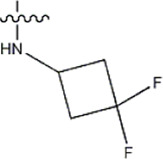	14	17b
	—	—	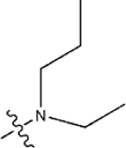	1.0	19e
	—	—	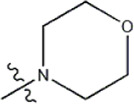	1.9	19f
	—	—	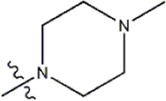	0.55	19g*
	—	—	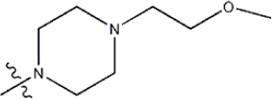	1.1	19h
	—	—	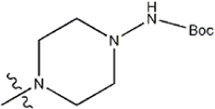	1.7	19i
	—	—	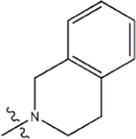	2.6	19j
	—	—	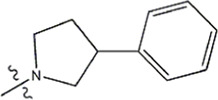	2.0	19k
	—	—	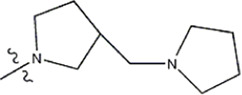	1.0	19l
	—	—	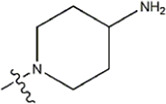	8.6	19m*
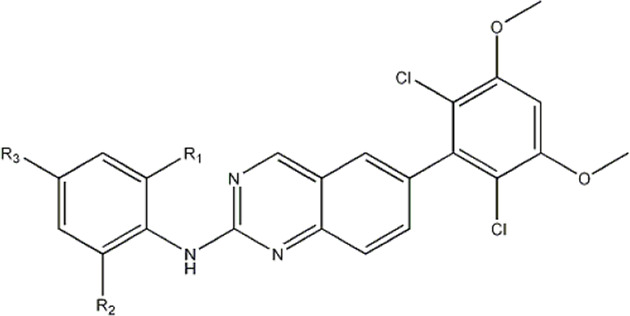	-NO_2_	-H	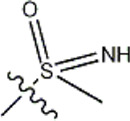	3577	33f
	-NH_2_	-H		516	33j
	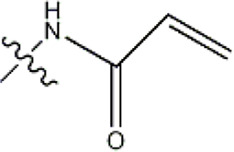	-H	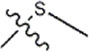	30	34a
		-CH_3_	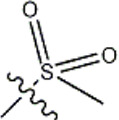	10	34b
		-H		33	34c
		-CH_3_	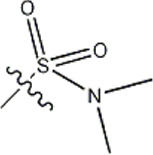	13	34d
		-H		50	34e
		-CH_3_	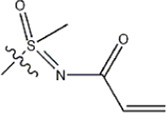	11	34f*
		-H		17	34g
		-CH_3_	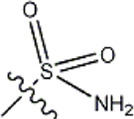	8.5	35a

Note: The underlined compound name in Table 1 represents the test set in the 2D-QSAR, experiment, and * represents the test set in the 3D-QSAR, experiment.

### 2.2 2D-QSAR research

#### 2.2.1 2D-QSAR data processing

In machine learning, to evaluate the discrimination, stability, robustness and other model effects of supervised algorithms, and to avoid over-fitting of data, it is necessary to divide the data set into a training set and a test set in the ratio of 4:1 (Mitchell et al., 1990).

In this experiment, system time was used as a random seed to split the dataset into a training set containing 29 compounds and a training set containing 8 compounds in a ratio of 4:1. The training set was used to train supervised models, fit models, adjust parameters, select modal variables, and make other choices for the algorithms. The test set is used to evaluate the effectiveness of the training model without changing the parameters and effects of the model. Usually, the decision to retrain the model or to choose another algorithm is based on whether the validated model is overfitted or underfitted.

#### 2.2.2 Calculation of the descriptions

In 2D-QSAR models, compounds are typically represented by molecular descriptors that can be statistically correlated with biological or even physicochemical properties. The molecular descriptors are calculated as follows: first, the molecular structures of 37 compounds were drawn in ChemDraw software. Then, to obtain stable molecular structures with the lowest energy, the molecular structures were optimized in HyperChem 7.5 software (Stewart, 1989) using successive MM + molecular mechanics force fields and the semi-empirical PM3 method (Ivanciuc, 1996), and then the optimized molecules were put into MOPAC6.0 software. Finally, the results were imported into CODESSA software (Katritzky et al., 2001) to calculate five classes of molecular descriptors: constitutional, geometrical, topological, electrostatic and quantum-chemical.

#### 2.2.3 Linear modeling (Cao and Lin, 2003)

Heuristic Method (HM) is the method of descriptor screening in the CODESSA software, the greatest advantage of which is that it allows a complete search for a large number of molecular descriptors without checking all possible combinations of parameters. Meanwhile, the method allows to build the best linear regression equation. The steps of HM are as follows.

a. Selection of 1-parameter descriptor. The square of correlation coefficient (*R* ^2^), F-test and *t*-test are used as selection criteria, and descriptors with low correlation with properties (activity) are removed. The descriptors without significant changes and those with high correlation are removed.
b. Selection of 2-Parameter Descriptor. *R* ^2^ and F- tests are the criteria for analysis and selection.
c. Selection of n-parameter descriptors. After obtaining the two-parameter correlation coefficients with optimal statistical characteristics, descriptors not used in the previous selection process are added to establish a new correlation equation. The new correlation coefficient is verified by *R* ^2^, F-test and standard deviation (S). Until the established correlation equation contains the maximum number of parameters. The process of adding descriptors starts with the correlation equation with the maximum fit-ness function value, which is defined as follows:

$$w=\left(R^{2}*F*n\right)/\left(N*S^{2}\right)①$$

where *R* ^2^ donates the square of the correlation coefficient, F is F-test value; n represents the number of samples, N is the number of descriptors and S is the standard deviation.

d. Output. Every time a descriptor is added, the optimal 10 correlation results are displayed, and every iteration starts with the results with the best correlation. The correlation between descriptors and the square of the cross-validation coefficient (R^2^_CV_) should be calculated during each cycle.

#### 2.2.4 Establishment of non-linear model

**FIGURE 1:**
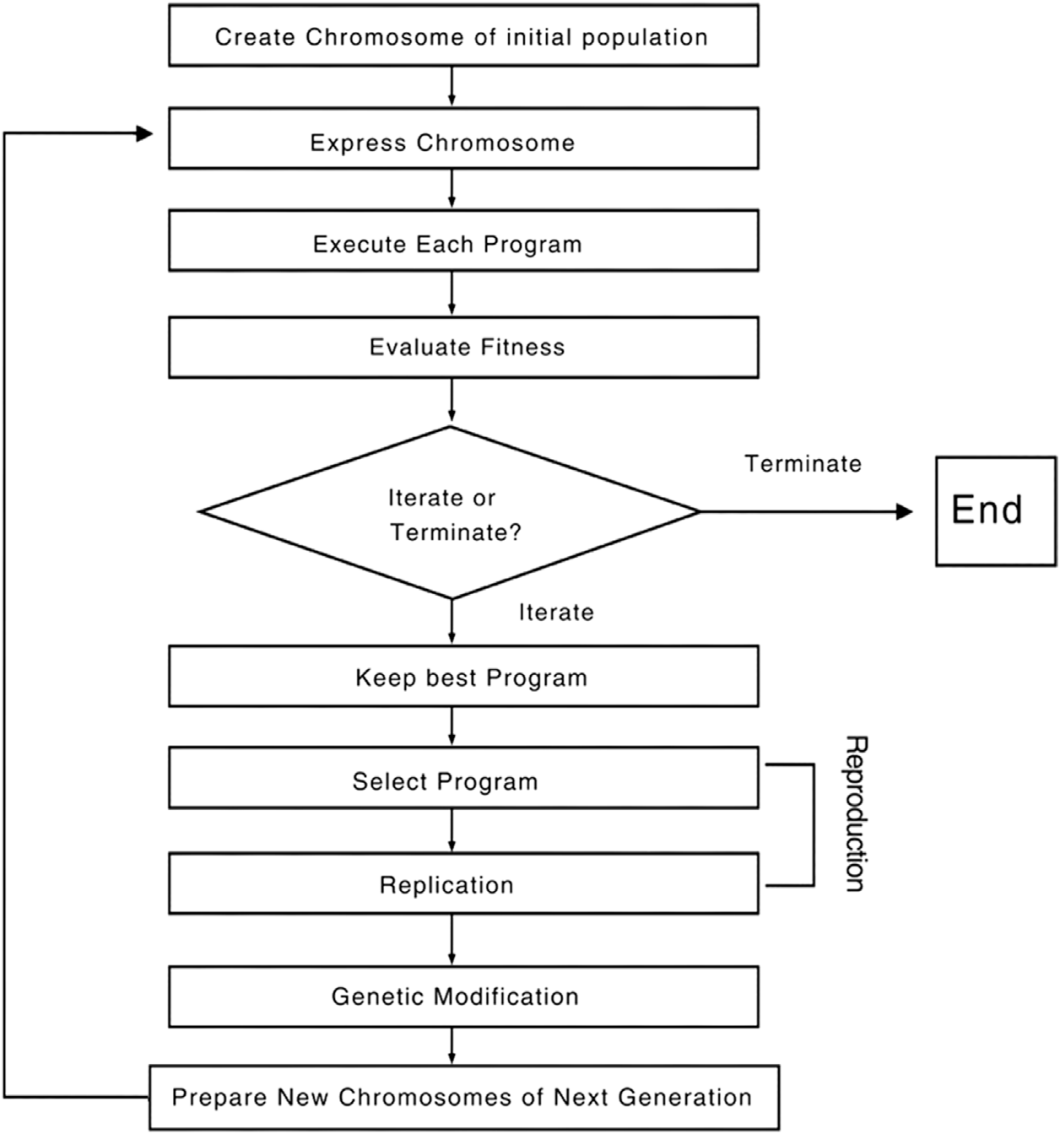
Flowchart of GEP algorithm.

GEP (Gene expression programming) is a new genetic algorithm that combines the advantages of genetic programming and genetic algorithm to solve complex problems with simple codes (Holland, 2005). According to the gene expression law of biological inheritance, GEP adopts equal-length linear symbols as the genetic code, and the individual phenotype as the expression tree. After a large number of operations, the algorithm can find the optimal solution. The process of GEP algorithm is described in detail as follows:

First, a certain number of chromosomes were randomly created as the initial population and then all chromosomes were translated into corresponding expression trees (ETs). Next, the fitness of each chromosome was measured according to a predefined fitness function to determine whether the fitness satisfies the termination criterion (a solution of the desired quality was found or a certain number of iterations had been completed). If the termination criteria were not met, the appropriate individuals were retained by an elitist roulette selection method. The selected individuals underwent genetic manipulation to form new individuals based on a certain probability, including variation, recombination and transposition. Finally, a new generation was created. Moreover, the chromosome of an individual consisted of one or more genes, represented by a fixed-size linear symbol string. The GEP gene consisted of two parts, which contain multiple gene element bits. The values of the gene element bits were taken from the set of terminal T and the set of function F (Teodorescu and Sherwood, 2008; Gharagheizi et al., 2012; Pham and Karaboga, 2012; Kaydani et al., 2014). Figure 1 summarizes the above operation process.

By comparing the linear model and non-linear model in 2D-QSAR model, it is found that the non-linear model generated by GEP algorithm is more reliable and has stronger prediction power than the linear model generated by HM method. However, the 2D-QSAR model cannot accurately describe the relationship between molecular 3D structure and physiological activity, so it is necessary to continue the 3D-QSAR experiment.

### 2.3 3D-QSAR research

#### 2.3.1 Data processing and structure optimization

In 3D-QSAR experiments, the IC_50_ values of all compounds need to be first converted to ′-log (IC_50_) +9′, which makes the data more stable and reduces the error caused by the original values in the experiment. Similar to 2D-QSAR experiment, the 3D-QSAR experiment also required randomly dividing the 37 quinazoline derivatives into training and test sets.

In the previous experiment, ChemDraw software was used to construct all 37 compounds, and in the 3D-QSAR experiment, these 37 compounds were put into SYBYL software for optimization and modeling. When processing data in SYBYL software, Tripos force field and Powell gradient algorithm were used to minimize COMSIA structure energy. Finally, the minimal structure was used as the initial conformation (Yu et al., 2015).

#### 2.3.2 Conformational sampling and alignment

**FIGURE 2:**
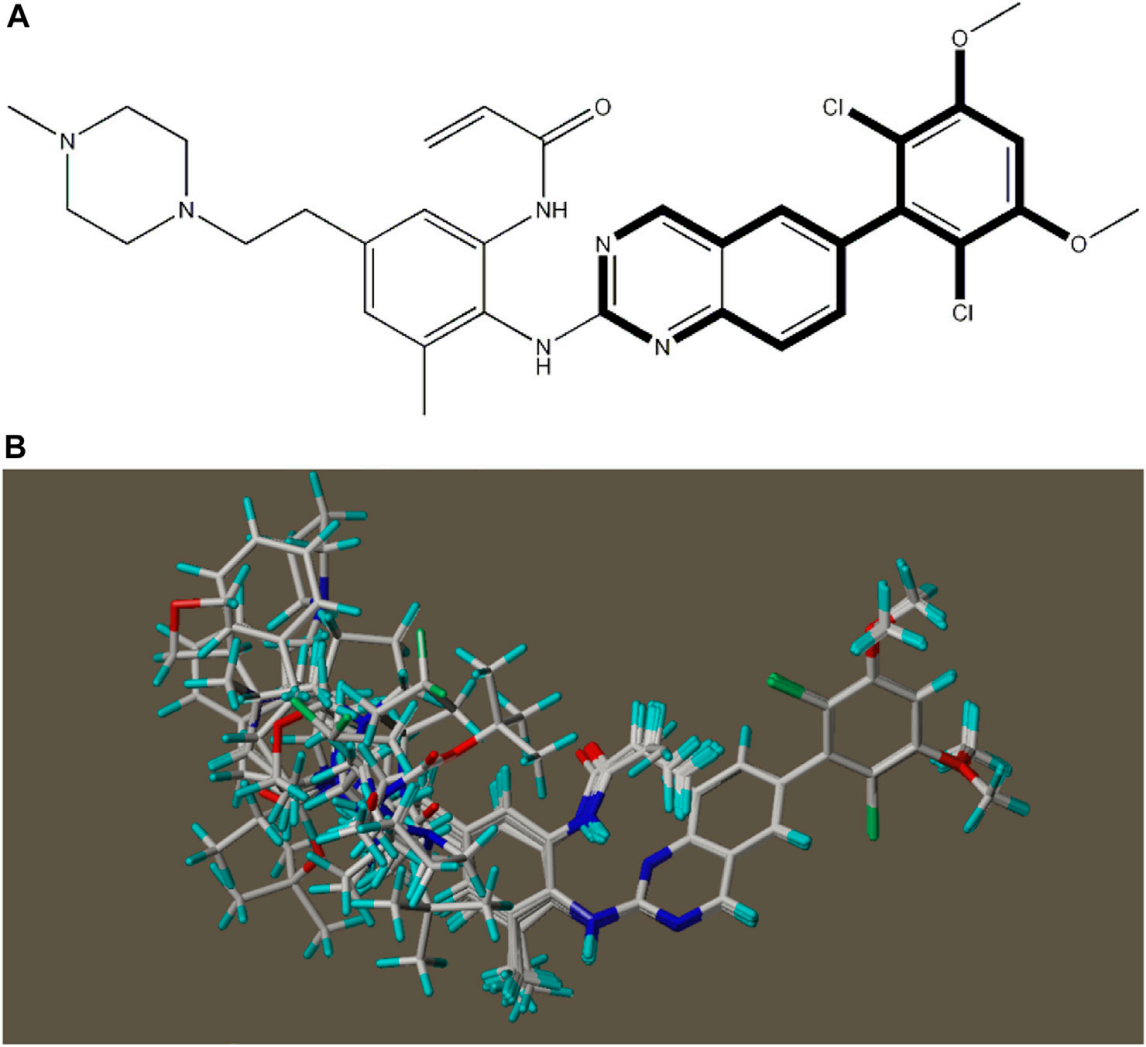
Compound 19g serves as a template for the alignment of all compounds. **(A)** Common alignment structures in compound 19g (shown in bold), and **(B)** all compounds are aligned with 19g as a template.

In 3D-QSAR experiments, the structure alignment of compounds will directly affect the establishment of subsequent 3D models, so it is very important to select appropriate structure alignment methods for compounds (Li et al., 2005; Patel et al., 2008; Ai et al., 2011). Ligand-based alignment was used in this experiment (Figure 2) since compound 19g was the compound with the best compound activity in the dataset, all compounds were aligned with compound 19g as a template.

#### 2.3.3 COMSIA study

In 1994, Klebe et al. H1 proposed the method of Comparative Molecular Similarity Index Analysis (CoMSIA), an extension of CoMFA. Both methods share the same principles and are based on the following assumptions when the bond affinity of a molecule changes, its corresponding molecular properties also change, which are expressed in the form of molecular fields (Yu et al., 2015).

In CoMSIA, the use of a distance-dependent Gaussian functional form for the calculation of various molecular fields effectively avoids significant changes in potential energy and anomalous positions of atoms at lattice points near the molecular surface. In addition, there is no need to define cut-off values for the energy in CoMSIA. Compared to CoMFA, CoMSIA corresponds to a significantly improved contour map of the contribution of different molecular fields in space, which allows a more intuitive interpretation of the effect of different molecular fields on molecular activity (Yang et al., 2011a; Li et al., 2012; Hadni and Elhallaoui, 2020).

For CoMSIA analysis, a grid with side lengths of 2 was first generated in all the assembled regions of the superimposed molecules, and the boundaries of 4Å were used to determine the regions of all the superimposed molecules. The default probe provided by CoMSIA was used to calculate the steric field, electrostatic field, hydrophobic field and hydrogen bond field (including hydrogen bond acceptor and hydrogen bond donor) at each grid point. After obtaining the molecular field for each grid, Partial Least Square (PLS) method was used to establish the quantitative correlation model between molecular field parameters and affinity. Finally, the Leave One Out (LOO) method and Cross Validation (CV) method were used to test the statistical significance of the model and determine the number of principal components of the model. The number of principal components determined by the optimal interaction verification value was used to establish the 3D-QSAR model without interaction verification. The affinity of the compounds in the test set was predicted (Yan et al., 2020).

#### 2.3.4 Validation of 3D-QSAR model

To demonstrate the stability of the QSAR model, the 3D-QSAR model needs to be evaluated using internal or external validation methods (Yan et al., 2020). In this experiment, external validation was selected to verify the 3D-QSAR model. The verification formula is as follows:

$$R_{ext}^{2}=1-\frac{\sum_{i=1}^{ntest}{\left(y_{i}-{\tilde{y}}_{i}\right)}^{2}}{{\left.\sum_{i=1}^{ntest}\left(y_{i}\right.-{\tilde{y}}_{tr}\right)}^{2}}②$$

where ntest refers to the number of compounds in the test set, ${\tilde{y}}_{tr}$ refers to the average value of compound activity in the training set, $y_{i}$ and ${\tilde{y}}_{i}$ , refer to the experimental value and predicted value of compound activity in the test set respectively. Generally, with $R_{ext}^{2}$ >0.5, the established model is considered to be robust and has good predictive ability in statistics (Yang et al., 2011b; Awasthi et al., 2018).

#### 2.3.5 Molecular docking experiment

After introducing the compound molecules into the Sybyl software, the molecular mechanics of the ligand small molecules were optimized using the Cong-Grad gradient method. A Tripos force field was used in the optimization process with energy convergence to 0.01 kcal/(mol-Å) and a maximum number of 106 iterations. After the optimization of molecular mechanics, the conformation with the lowest energy was selected for further molecular docking studies. For the FGFR4 receptor (crystal structure of the protein from the RCSB Protein Data Bank, PDB ID:4xcu), crystal water molecules and hydrogenated atoms were removed and the original ligands in the protein were extracted to identify their binding sites for subsequent molecular docking.

Flexible docking between small molecule ligands and receptors was performed using Sybyl software. The ligand binding sites were used to generate target active pockets. Docking was subsequently performed using the Sybyl-Dock standard model with a threshold parameter of 0.5 and an expansion factor of 1. Molecular conformational changes were retained by 20. Total-Score function of the Sybyl-Dock module was used to score the interaction between the small molecule and the target, taking into account the effects of polarity, hydrophobicity and enthalpy. The higher the value, the better the interaction between the compound molecule and the protein crystal.

## 3 Results and discussion

### 3.1 HM

**FIGURE 3:**
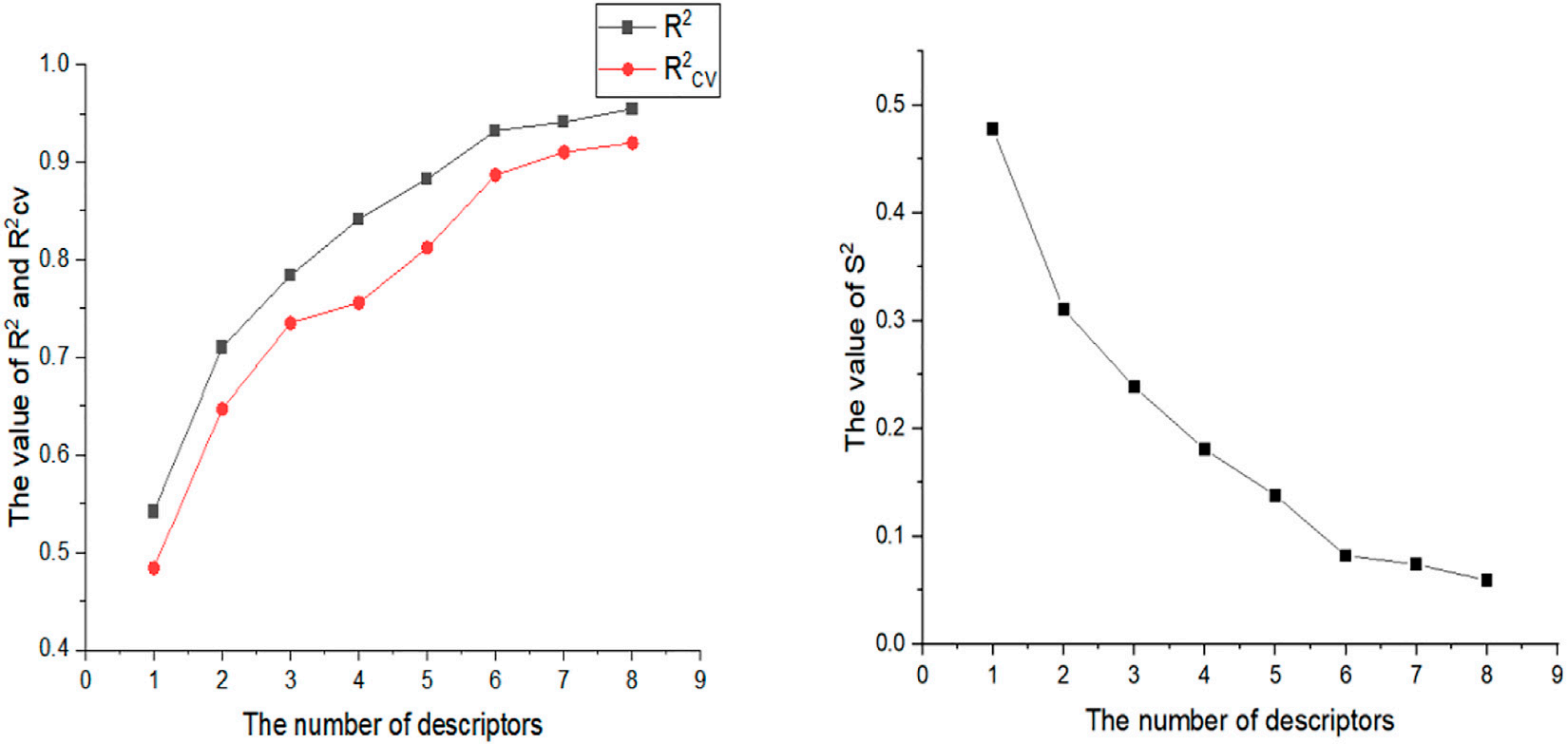
The effects of different numbers of descriptors on *R* ^2^, R^2^cv and S^2^.

**FIGURE 4:**
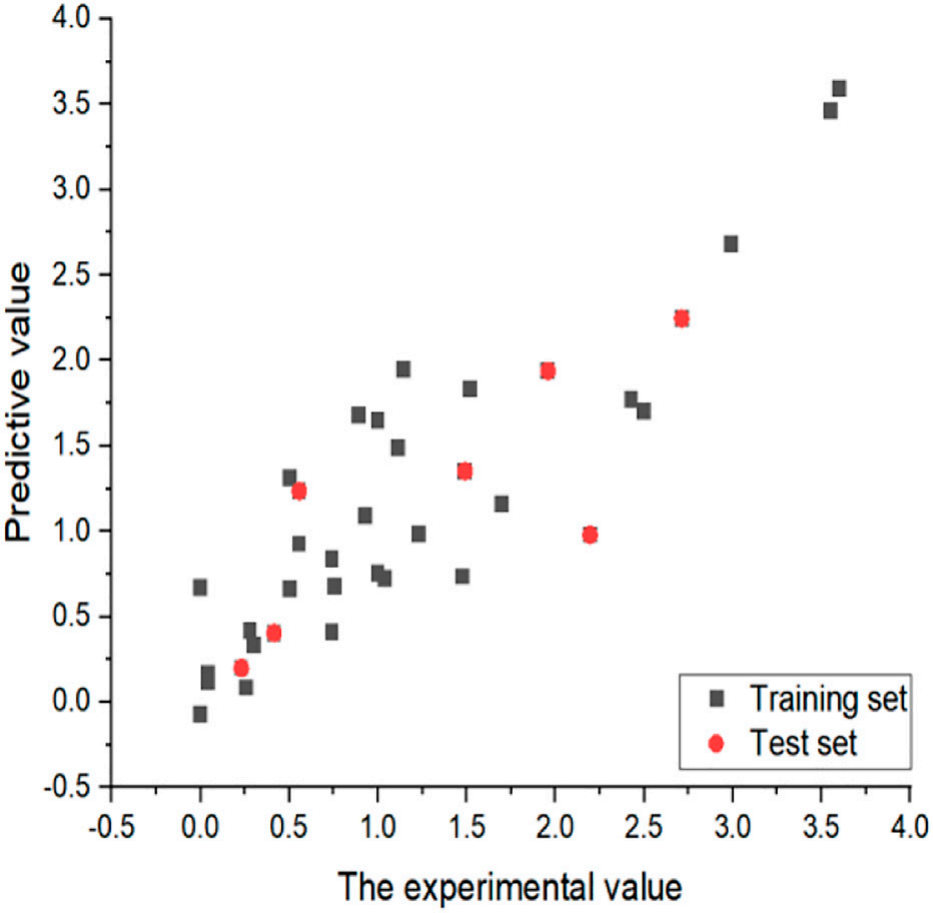
Plots of measured and calculated log(IC_50_) by HM.

In this experiment, 593 descriptors for 37 compounds were calculated using CODESSA software. Also, to obtain a set of descriptors most relevant to the activity of osteosarcoma inhibitors, eight linear regression QSAR models were developed using the HM method with the number of molecular descriptors ranging from 1 to 8, where the effects of different numbers of descriptors on *R* ^2^, R^2^cv and S^2^ is shown in Figure 3. The results show that the number of descriptors is proportional to the values of *R* ^2^ and R^2^cv, and inversely proportional to S^2^. The model with three descriptors was selected as the best linear model for predicting the activity of osteosarcoma receptor inhibitors. Table 2 shows the details of these descriptors. In addition, to ensure that there is no multicollinearity between the molecular descriptors calculated in this experiment, the correlation coefficients between descriptors were calculated by CODESSA software, as shown in Table 3. The correlation coefficient between any two descriptors is less than 0.8, indicating that all descriptors are independent. Therefore, the constructed linear model has strong statistical reliability. The HM model is illustrated in Figure 4.

**TABLE 2:** Details of the three selected descriptors.

Symbol	Physical-chemical meaning	Coefficient	*t*-test
Avg-ERC	Avg electroph react index for a C atom	1.5952e+03	8.6342
HDSA-2/TMSA(QC)	HA dependent HDSA-2/TMSA [Quantum-Chemical PC]	−1.1630e+02	−4.6161
Min-NRO	Min nucleoph react index for an O atom	2.5642e+04	3.4256

**TABLE 3:** Correlation coefficient between three descriptors.

Name	Avg-ERC	HDSA-2/TMSA(QC)	Min-NRO
1	1.0000	0.0220	0.1367
2	0.0220	1.0000	−0.1066
3	0.1367	−0.1066	1.0000

The linear model formula is as follows:

$$\mathrm{Log}\left(IC50\right)=1.5952*103*\;Avg-ERC-1.1630*102*\;HDSA-2/TMSA\left(QC\right)+2.5642*104*\;\mathrm{Min}-NRO-7.5167*10-1$$

From the coefficients of the three descriptors in the above formula, it can be seen that the absolute value of the coefficient of the descriptor “Min-NRO” is the largest, so the descriptor “Min-NRO” has the greatest influence on the linear model of this experiment.

### 3.2 GEP

The construction of non-linear model in this experiment is mainly realized by the GEP algorithm in APS software. All the functions used in the operation of GEP algorithm are shown in Table 4. Finally, a satisfactory non-linear model was obtained when GEP was used for 220 generations. The correlation coefficients of the training and testing sets of the non-linear model were 0.89 and 0.86, respectively, with average errors of 0.02 and 0.04.

**TABLE 4:** All operation functions of GEP algorithm.

Parameter name	Representation	Values
Addition	+	1
Subtraction	-	1
Multiplication	*	1
Division	—	1
Cosine	Cos(x)	1

In addition, the non-linear model equation obtained by GEP algorithm (converted by C language) is as follows:

$$\mathrm{Log}\left(IC50\right)=14y+2x+1+\cos\left(y/3x-\cos\left(x\right)\right)+\cos\left(\cos\left(x*x/y/z*\cos\left(2y\right)\right)\right)+y/3x-\cos\left(1\right)$$

where x, y and z represent the descriptors Avg-ERC, HDSA-2/TMSA(QC) and Min-NRO, respectively.

**FIGURE 5:**
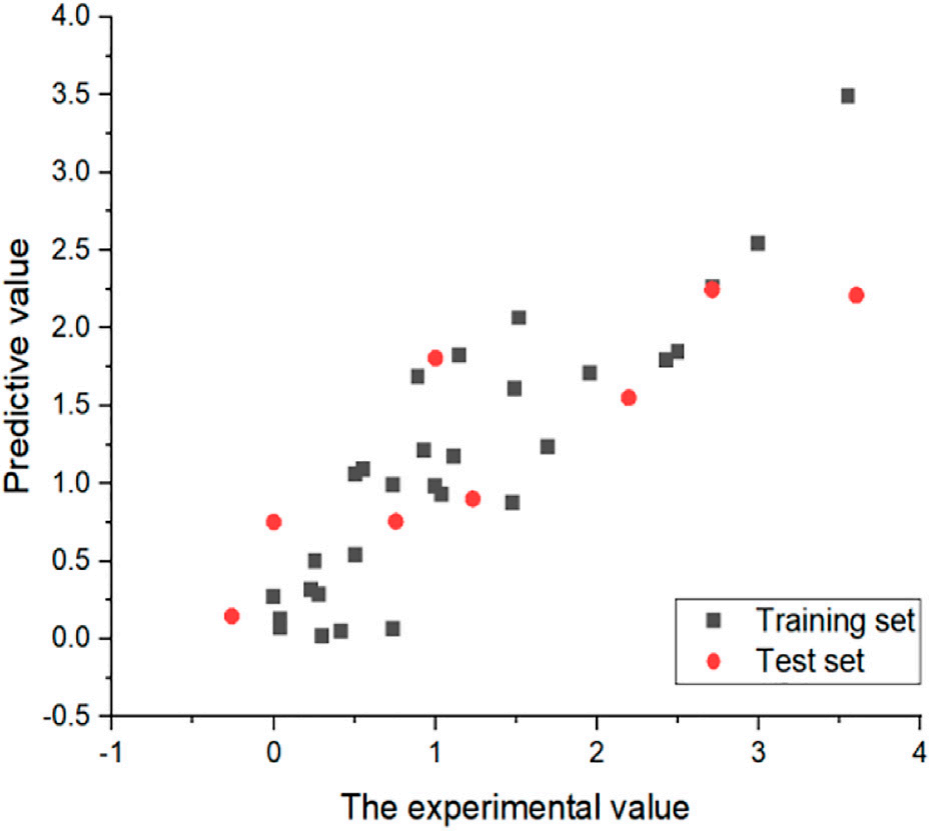
Experimental and predicted values for non-linear models. All IC_50_ values are converted to log(IC_50_).

In order to verify the predictive ability of the non-linear model, the formula was used to predict the IC_50_ value of all compounds. The specific results are shown in Figure 5, indicating that the predictive ability of the non-linear model is extremely reliable.

### 3.3 COMSIA model results

In the 3D-QSAR experiments, eight groups of COMSIA models were obtained by permuting and combining the steric field (S), electrostatic field (E), hydrophobic (H), hydrogen bond donor field (D) and hydrogen bond acceptor field (A). Among them, the model composed of SEHDA has the best statistical results, and the details are shown in Table 5.

**TABLE 5:** Statistical results of the optimal CoMSIA model.

Model	q^2^	ONC	*r* ^2^	SEE	F
CoMSIA	0.63	6	0.987	0.056	189.027

Name	S	E	H	D	A
Contribution (%)	6.2	29.6	18.6	19.9	25.8

Notes: ONC, the optimum number of components. SEE, the standard error of estimate. S, steric. E, electrostatic. H, hydrophobic. D, hydrogen bond donor. A, hydrogen bond acceptor.

### 3.4 External verification results

**FIGURE 6:**
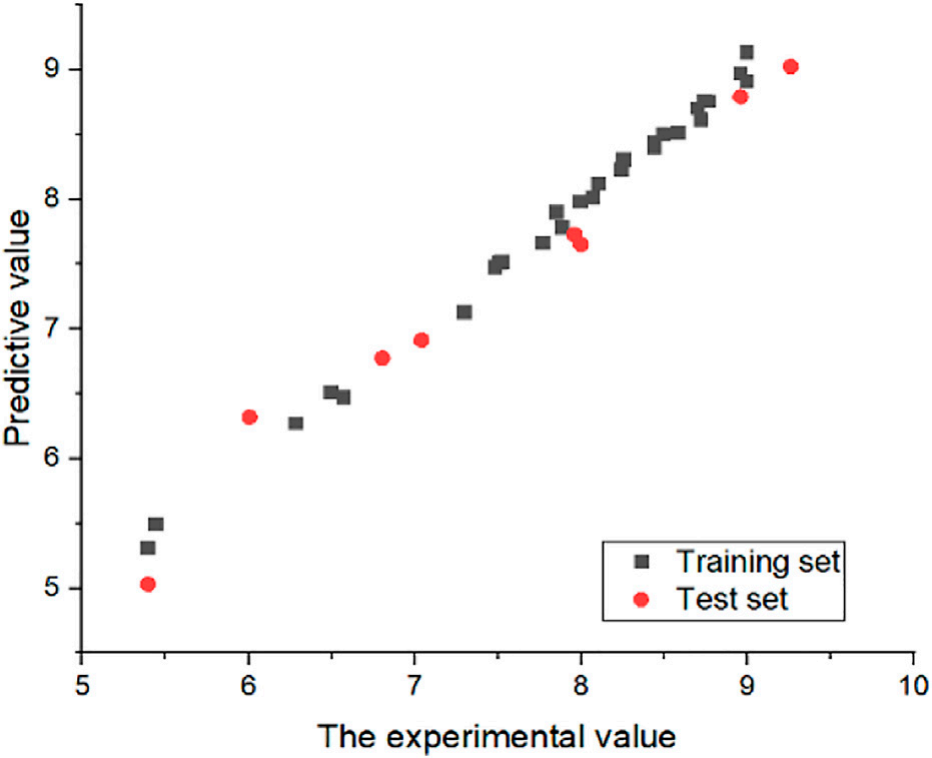
Experimental and predicted values of CoMSIA model. All compounds IC_50_ were converted to “−log(IC_50_)+9.”

In order to prove the stability of the 3D-QSAR model constructed in this experiment, the external verification formula was used to verify the model with satisfactory results. The value of $R_{ext}^{2}$ obtained by external verification formula was 0.997, greater than 0.5, indicating that the CoMSIA model constructed in this experiment has a strong predictive ability. At the same time, in order to further demonstrate the predictive ability of the model, all compounds were put into the model to predict their compound activity, which can be found to be extremely reliable (Figure 6).

### 3.5 COMSIA contour maps

One of the advantages of CoMSIA is the ability to isolate and observe the effects of various physical and chemical properties on biological activity through 3D correlation contour maps. This contour map helps to identify the important regions of the molecular field that affect the biological activity, and also to mark the molecular field features that contribute significantly to the active site of ligands and receptors (Chen et al., 2021; Wang et al., 2022).

**FIGURE 7:**
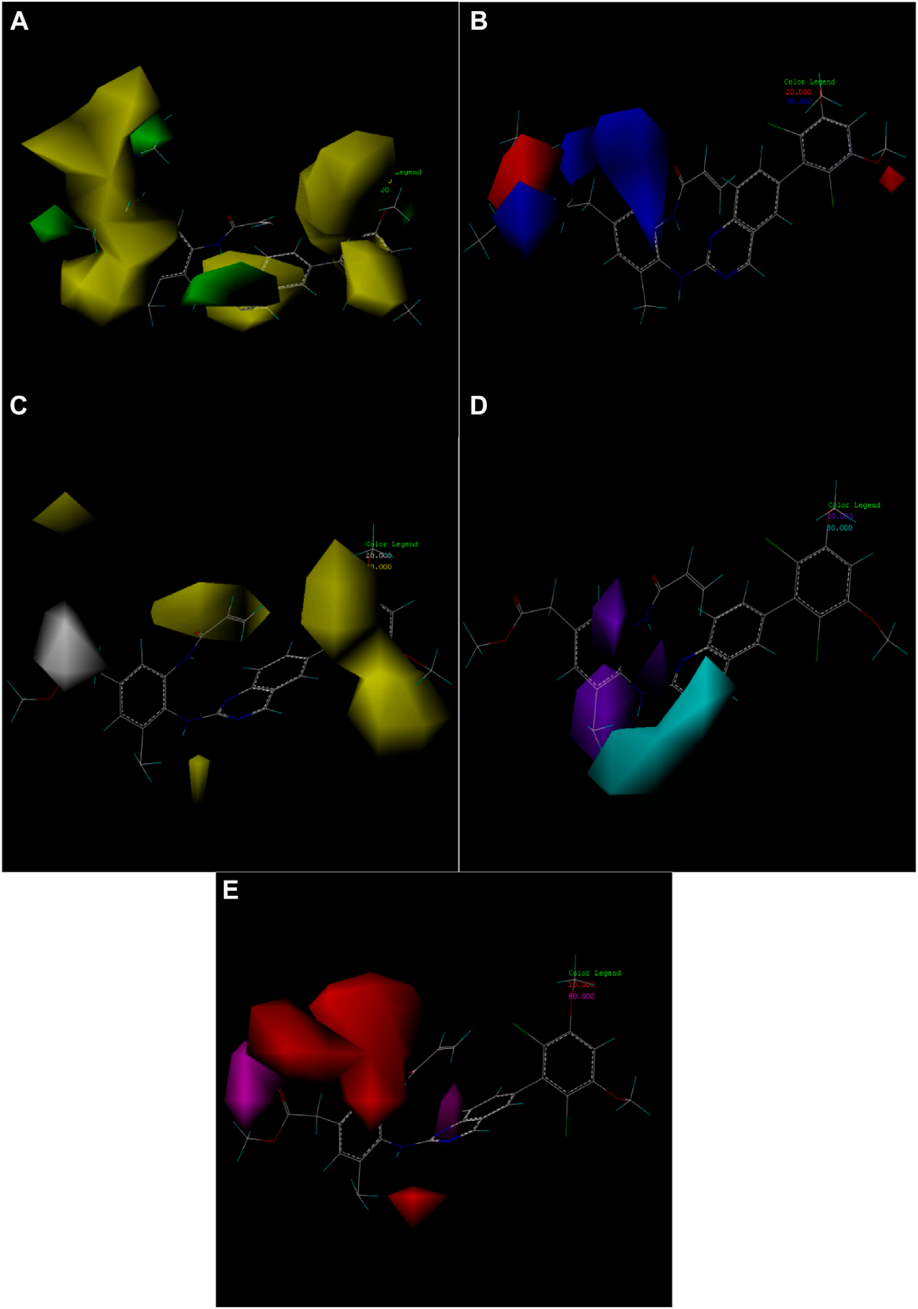
Contour map of optimal compound 19g. **(A)** In the steric field, green represents favorable and yellow represents unfavorable. **(B)** In the electrostatic field, blue represents a positive electric field and red represents a negative electric field. **(C)** In the hydrophobic field, yellow represents favorable and white represents unfavorable. **(D)** Favorable (cyan) and unfavorable (purple) hydrogen bond donor fields. **(E)** Favorable (magenta) and unfavorable (red) hydrogen bond acceptor fields.

In this experiment, the steric, electrostatic, hydrophobic, hydrogen bond donor and acceptor fields were constructed according to the compound 19g with the best compound activity, among which the electrostatic field had the highest contribution. In other words, the influence of electrostatic field on the compound activity should be taken into consideration in subsequent compound design experiments. Figure 7 shows the five contour maps.

### 3.6 Design new compounds and predict activity

From the results of 2D-QSAR experiments, we know that the descriptor ‘Min nucleoph React index for an O Atom' has the greatest impact on the compound activity of compounds. Fukui atomic nucleophilic reaction index formula (Franke, 1984) has a very detailed explanation for this kind of descriptors, as expressed as follows:

$$N_{A}=\underset{i\in A}{\sum }C_{iHOMO}^{2}③$$

where $C_{iHOMO}^{2}$ stands for highest occupied molecular orbital MO coefficients, $N_{A}$ stands for minimum nucleophilic reaction index. Through an in-depth study of this formula, it is easy to conclude that the regression coefficient of “Min nucleophilic reaction index of O atom” is positive, and the more negatively charged oxygen atoms in the molecule, the greater the nucleophilic reaction index. The larger the nucleophilic reaction index, the larger the IC_50_ value of the compound, and the lower the compound activity. According to this conclusion, the activity of a compound can be improved by increasing the valence of the oxygen atom or decreasing the number of oxygen atoms in the compound.

Electrostatic field contributed the most in the five CoMSIA contour maps, so electrostatic field is the main factor to be considered in compound design. Of course, there are other force fields that need to be considered. The biggest innovation of this study is the design of new compounds by combining the descriptor “minimum nuclear reaction index of O atoms” and CoMSIA contour maps. The specific combination method is to reduce the number of molecular oxygen atoms or increase the valence of oxygen atoms in the favorable region of contour map according to Fukui nucleophilic reaction formula. Finally, 200 new quinazolines were designed. Table 6 shows the ten compounds with the highest compound activity. Compound 19g.10 has the highest value of antitumor activity among these compounds and can be considered as a potential chemotherapeutic agent for the treatment of osteosarcoma. However, in order to prove the target binding capability of the newly designed compounds with osteosarcoma targets, it is necessary to continue with small molecule docking experiments.

**TABLE 6:** The compounds of newly designed and their predicted values.

Name	Predictive value
19g	9.027
19g.1	10.135
19g.2	10.178
19g.3	10.289
19g.4	10.336
19g.5	10.545
19g.6	10.651
19g.7	10.661
19g.8	10.674
19g.9	10.845
19g.10	10.901

### 3.7 Molecular docking results

**FIGURE 8:**
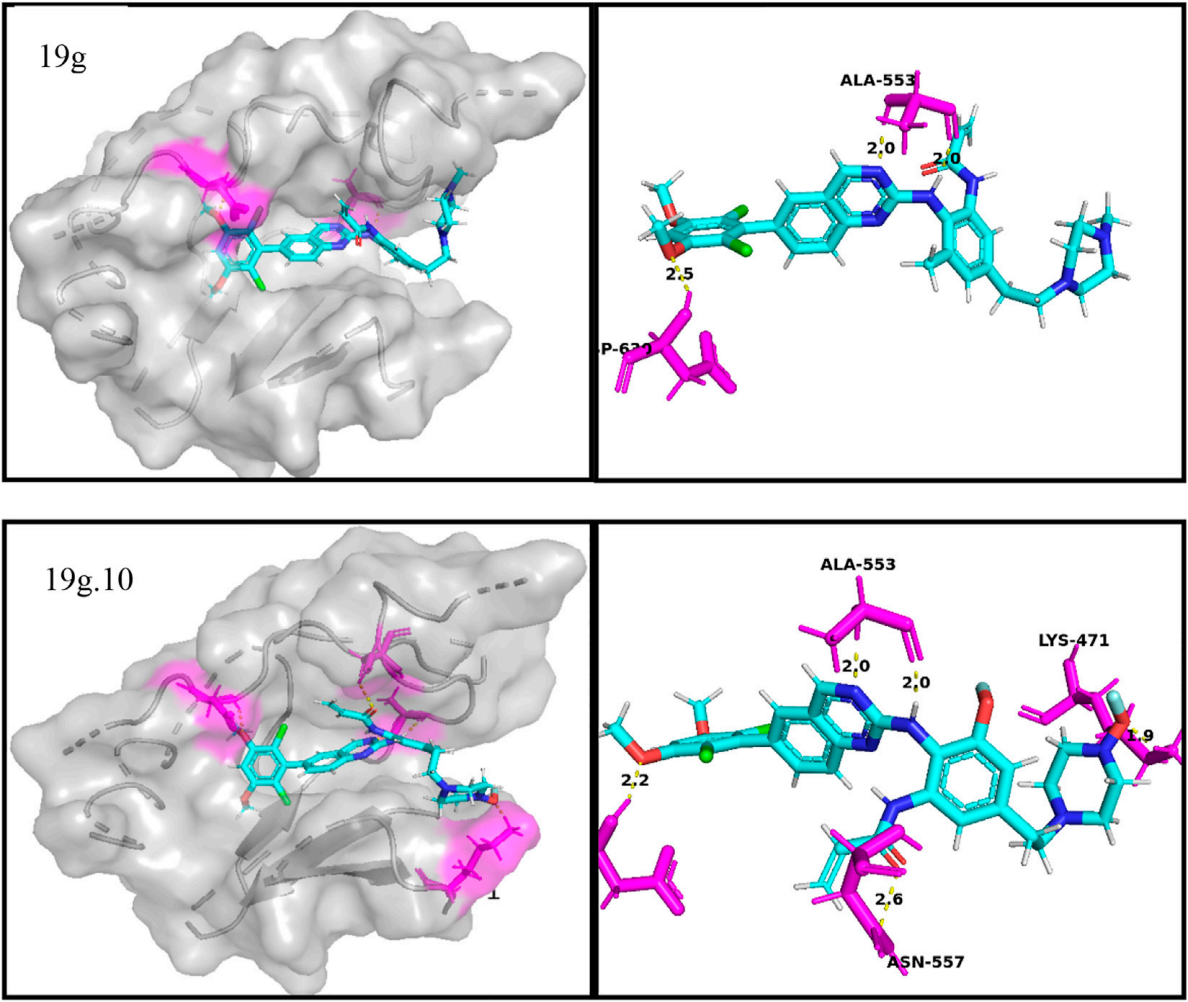
Docking assay of compounds 19g, and 19g.10 with osteosarcoma related target (FGFR4, PDB ID: 4XCU).

In docking experiments, compound 19g and the new compound 19g.10 were used as ligands for docking with the osteosarcoma-related target FGFR4, and Figure 8 represents the docking results for both compounds (yellow dashed lines represent hydrogen bonds).

From the docking conformation of compound 19g, the oxygen atom (located in the structure 2,4-Dichloro-1,5-dimethoxy-3-methylbenzene) formed a hydrogen bond with the protein residue ASP630, and the nitrogen atom of the quinazoline core, as well as the hinge region, formed a hydrogen bond with Ala553. This is the same binding mode as that of BLU9931, a selective inhibitor of FGFR4 first identified by Hagel M, Miduturu C et al. (Hagel et al., 2015).

From the docking conformation of compound 19.10, it appears that compound 19g.10 can form hydrogen bonds not only with residues ASP630 and ALA553 but also the N-methylpropanamide structure of the compound can form hydrogen bonds with residue ASN557. And the 1-fluoride-4-methylpiperazine structure can also form hydrogen bonds with the protein residue LYS471. The above results suggest that compound 19g.10 designed in this experiment does have potential as a chemotherapeutic candidate for osteosarcoma.

## 4 Conclusion

In this study, linear and non-linear 2D-QSAR models were obtained experimentally, and it can be seen from the experimental results that the descriptor Min-NRO has the greatest influence on compound activity. However, it is obvious that only 2D experiments are not enough, so it is necessary to continue with 3D conformational relationship experiments. Through the CoMSIA method, a 3D-QSAR model with high q^2^ (0.63) and *r* ^2^ (0.987) values and low error values (0.05) was obtained. In addition, among the five contour maps of CoMSIA model, the contour map that contributes most to the compound activity of the compound is the electrostatic field.

Finally, 200 new quinazoline derivatives were designed according to the molecular descriptor Min-NRO and contour map. In order to further verify the correlation between the compounds and the targets of osteosarcoma, small molecule docking experiments were also performed in this study, indicating that compound 19g.10 also had good target binding capability and can be regarded as a potential target compound for osteosarcoma. In conclusion, this study designed a reliable QSAR model for quinazoline compounds, which provided new ideas for compound design and could provide important guidance for the development of future chemotherapeutic drugs for osteosarcoma.

## Data Availability

The original contributions presented in the study are included in the article/supplementary material, further inquiries can be directed to the corresponding author.
